# Endogenous sulfur dioxide is a novel adipocyte-derived inflammatory inhibitor

**DOI:** 10.1038/srep27026

**Published:** 2016-06-01

**Authors:** Heng Zhang, Yaqian Huang, Dingfang Bu, Selena Chen, Chaoshu Tang, Guang Wang, Junbao Du, Hongfang Jin

**Affiliations:** 1 Department of Endocrinology, Beijing Chaoyang Hospital, Capital Medical University, Beijing, China; 2 Department of Pediatrics, Peking University First Hospital, Beijing 100034, China; 3 Research Center, Peking University First Hospital, Beijing 100034, China; 4 University of California, San Diego, La Jolla, California, 92093, United States of America; 5 Department of Physiology and Pathophysiology, Peking University Health Science Center, Beijing 100191, China; 6 Key Lab. of Ministry of Education of China, Beijing, China

## Abstract

The present study was designed to determine whether sulfur dioxide (SO_2_) could be endogenously produced in adipocyte and served as a novel adipocyte-derived inflammatory inhibitor. SO_2_ was detected in adipose tissue using high-performance liquid chromatography with fluorescence detection. SO_2_ synthase aspartate aminotransferase (AAT1 and AAT2) mRNA and protein expressions in adipose tissues were measured. For *in vitro* study, 3T3-L1 adipocytes were cultured, infected with adenovirus carrying AAT1 gene or lentivirus carrying shRNA to AAT1, and then treated with tumor necrosis factor-α (TNF-α). We found that endogenous SO_2_/AAT pathway existed in adipose tissues including perivascular, perirenal, epididymal, subcutaneous and brown adipose tissue. AAT1 overexpression significantly increased SO_2_ production and inhibited TNF-α-induced inflammatory factors, monocyte chemoattractant protein-1 (MCP-1) and interleukin-8 (IL-8) secretion from 3T3-L1 adipocytes. By contrast, AAT1 knockdown decreased SO_2_ production and exacerbated TNF-α-stimulated MCP-1 and IL-8 secretion. Mechanistically, AAT1 overexpression attenuated TNF-α-induced IκBα phosphorylation and degradation, and nuclear factor-κB (NF-κB) p65 phosphorylation, while AAT1 knockdown aggravated TNF-α-activated NF-κB pathway, which was blocked by SO_2_. NF-κB inhibitors, PDTC or Bay 11-7082, abolished excessive p65 phosphorylation and adipocyte inflammation induced by AAT1 knockdown. This is the first report to suggest that endogenous SO_2_ is a novel adipocyte-derived inflammatory inhibitor.

Chronic inflammation in adipose tissue is considered to play a vital role in the pathogenesis of metabolic diseases, such as in obesity, insulin resistance and atherosclerosis^1^. Secretion of large amounts of pro-inflammatory cytokines such as monocyte chemoattractant protein-1 (MCP-1) and interleukin-8 (IL-8) from adipose tissue increased infiltration of local immune cells, aggravated chronic inflammation in adipose tissue, leading to adipose tissue dysfunction and metabolic disorders. Indeed, obese animals with a deficiency of MCP-1 or its receptor, chemokine (C-C motif) receptor 2 or CCR2, showed fewer macrophages infiltration in adipose tissue, attenuated local inflammation, and improved insulin sensitivity compared with the obese littermates^2^.

The endogenous gaseous signaling molecules participate in regulating the occurrence and development of cardiovascular diseases, nervous system diseases, gastric ulceration, and infection. Recent studies showed that endogenous hydrogen sulfide (H_2_S) participated in the pathogenesis of diabetes and might be a novel insulin resistance regulator^3^,^4^. Sulfur dioxide (SO_2_), a newly discovered gasotransmitter candidate, could be endogenously generated in cardiovascular system by the metabolism of sulfur-containing amino acids^5^. However, whether sulfur dioxide is endogenously generated in adipose tissue and whether it plays a role in regulating inflammatory factors secreted from adipocytes have not been explored. Therefore, the present study was designed to determine whether endogenous SO_2_ was generated in adipose tissue and to explore the role of SO_2_ played in the regulation of inflammatory factors secretion in adipocytes.

## Results

### Endogenous SO_2_/AAT pathway existed in adipose tissue of rats

**Figure 1:**
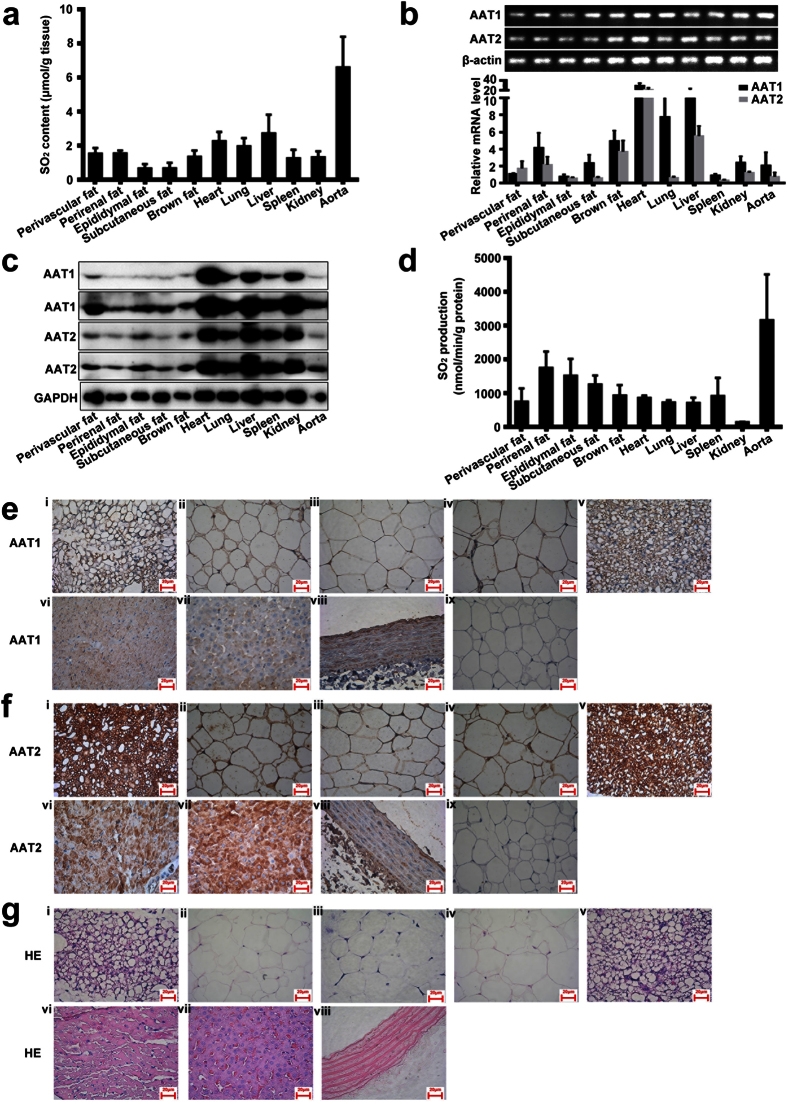
The AAT/SO_2_ system in rat adipose tissues (mean ± SEM). (**a**) SO_2_ content in rat tissue homogenate (perivascular adipose tissue, perirenal adipose tissue, epididymal adipose tissue, subcutaneous adipose tissue, brown adipose tissue, heart, lung, liver, spleen, kidney and aorta) by HPLC-FD. (**b**) RT-PCR analysis of AAT1 and AAT2 mRNA levels in rat tissue (perivascular adipose tissue, perirenal adipose tissue, epididymal adipose tissue, subcutaneous adipose tissue, brown adipose tissue, heart, lung, liver, spleen, kidney and aorta). (**c**) Western blot analysis of AAT1 and AAT2 protein expression in rat tissue homogenate (perivascular adipose tissue, perirenal adipose tissue, epididymal adipose tissue, subcutaneous adipose tissue, brown adipose tissue, heart, lung, liver, spleen, kidney and aorta). The bands of AAT1 and AAT2 were exposed twice. (**d**) Measurement of SO_2_ production from different rat tissues by addition of L-cysteine plus pyridoxal 5′-phosphate to tissue homogenate and incubation for 90 min. (**e**) Expression of AAT1 in different rat tissues using immunohistochemistry: i, perivascular adipose tissue; ii, perirenal adipose tissue; iii, epididymal adipose tissue; iv, subcutaneous adipose tissue; v, brown adipose tissue; vi, heart; vii, liver; viii, aorta; and ix IgG as a negative control. (**f**) Expression of AAT2 in different rat tissues using immunohistochemistry: i, perivascular adipose tissue; ii, perirenal adipose tissue; iii, epididymal adipose tissue; iv, subcutaneous adipose tissue; v, brown adipose tissue; vi, heart; vii, liver; viii, aorta; and ix IgG as a negative control. (**g**) Hematoxylin and eosin (HE) staining of different rat tissues: i, perivascular adipose tissue; ii, perirenal adipose tissue; iii, epididymal adipose tissue; iv, subcutaneous adipose tissue; v, brown adipose tissue; vi, heart; vii, liver; and viii aorta.

We detected the concentration of SO_2_ in respective rat adipose tissues, including perivascular, adipose tissue (1.53 ± 0.33 μmol/g protein), perirenal adipose tissue (1.54 ± 0.17 μmol/g protein), epididymal adipose tissue (0.65 ± 0.26 μmol/g protein), subcutaneous adipose tissue (0.67 ± 0.32 μmol/g protein), and brown adipose tissue (1.34 ± 0.37 μmol/g protein) (Fig. 1a). The content was comparable to that in the spleen and kidney, but lower than that in the heart, lung, liver and aorta (Fig. 1a).

SO_2_ generation in mammals mainly depends on two enzymes AAT1 and AAT2. RT-PCR revealed that both AAT1 and AAT2 mRNA were expressed in perivascular, perirenal, epididymal, subcutaneous and brown adipose tissue, with heart, lung, liver, spleen, kidney and aorta used as a positive control (Fig. 1b). Moreover, AAT1 and AAT2 protein expressions were also detected in respective rat adipose tissues by western blot analysis (Fig. 1c). The evaluation by the production of SO_2_ in the presence of L-cysteine in the adipose tissue homogenate showed that AAT activities were 747.37 ± 227.09 nmol/min/g in perivascular adipose tissue, 1745.18 ± 279.90 nmol/min/g in perirenal adipose tissue, 1516.22 ± 286.60 nmol/min/g in epididymal adipose tissue, 1253.75 ± 152.93 nmol/min/g in subcutaneous adipose tissue, and 927.41 ± 179.24 nmol/min/g in brown adipose tissue (Fig. 1d). Protein locations of AAT1 and AAT2 in different adipose tissues stained by immunohistochemical analysis are shown in Fig. 1e–g.

### AAT1 overexpression inhibited TNF-α-induced MCP-1 and IL-8 secretion in 3T3-L1 adipocytes

**Figure 2:**
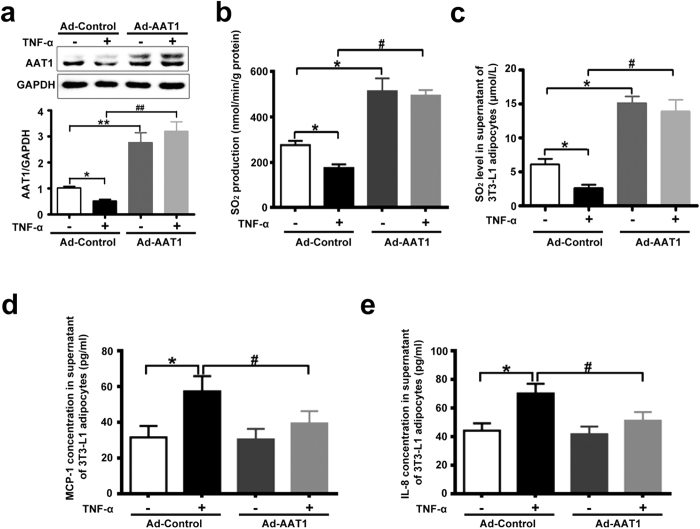
AAT1 overexpression decreased MCP-1 and IL-8 secretion from TNF-α-induced 3T3-L1 adipocytes. (**a**) Protein expression of AAT1 in 3T3-L1 adipocytes infected with AAT1 adenovirus (Ad-AAT1, 100 moi) or Ad-Control. (**b**) SO_2_ production in 3T3-L1 adipocytes. (**c**) SO_2_ level in supernatant of 3T3-L1 adipocytes infected with Ad-AAT1 or Ad-Control by HPLC-FD. (**d,e**) MCP-1 (**d**) and IL-8 (**e**): relative concentration in supernatant from 3T3-L1 adipocytes by ELISA. Adipocytes were infected with Ad-Control or Ad-AAT1 for 3 d, and then stimulated with TNF-α (10 ng/ml) for 2 h. Data are means ± SEM of 6 independent experiments. *P < 0.05 compared with Ad-Control group; **P < 0.01 compared with Ad-Control group; ^#^P < 0.05 compared with Ad-Control + TNF-α group; ^##^P < 0.01 compared with Ad-Control + TNF-α group.

Chronic inflammation in adipose tissue participated in the pathogenesis of insulin resistance and type 2 diabetes. To explore the effect of endogenous SO_2_ on MCP-1 and IL-8 secretion in 3T3-L1 adipocytes, 3T3-L1 adipocytes were infected with adenovirus carrying SO_2_ synthase AAT1, and then stimulated with TNF-α (10 ng/ml) for 2 h. TNF-α reduced protein expression of AAT1, the rate of SO_2_ production and SO_2_ concentration in 3T3-L1 adipocytes infected with control adenovirus (Ad-Control) (Fig. 2a–c). Infection of 3T3-L1 adipocytes with AAT1 adenovirus (Ad-AAT1) at 100 multiplicities of infection (moi) markedly increased AAT1 protein level, the rate of SO_2_ production and SO_2_ concentration compared with Ad-Control-infected 3T3-L1 adipocytes (Fig. 2a–c). ELISA analysis showed that MCP-1 and IL-8 concentrations in the supernatant were much higher in 3T3-L1 adipocytes treated with TNF-α for 2 h than in untreated adipocytes (Fig. 2d,e). AAT1 overexpression significantly decreased MCP-1 and IL-8 concentrations in supernatant from TNF-α-stimulated 3T3-L1 adipocytes (Fig. 2d,e).

### AAT1 knockdown exacerbated TNF-α-induced MCP-1 and IL-8 secretion in 3T3-L1 adipocytes

**Figure 3:**
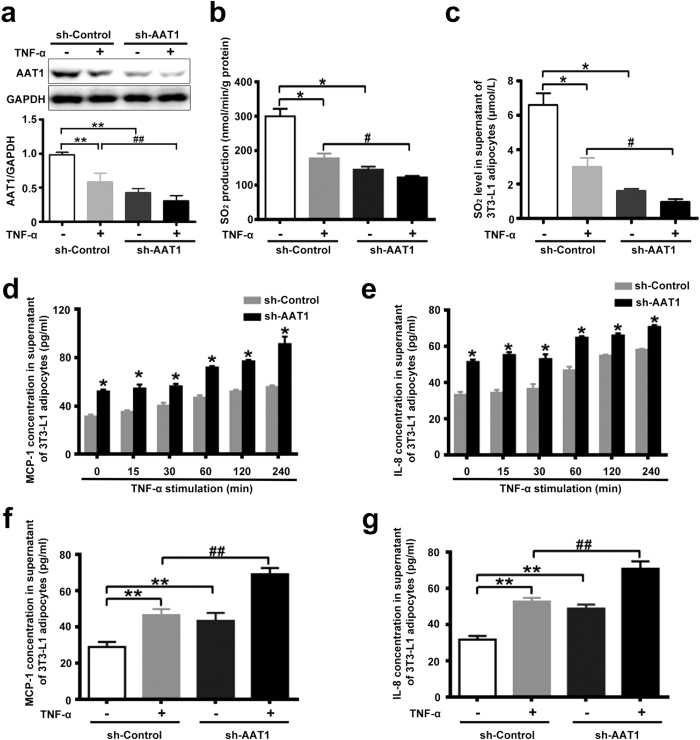
AAT1 knockdown exacerbated MCP-1 and IL-8 secretion from TNF-α-stimulated 3T3-L1 adipocytes. (**a**) Protein expression of AAT1 in 3T3-L1 adipocytes infected with lentivirus carrying shRNA to AAT1 (sh-AAT1, 100 moi) or sh-Control. (**b**) SO_2_ production in 3T3-L1 adipocytes. (**c**) SO_2_ level in supernatant of 3T3-L1 adipocytes infected with sh-AAT1 or sh-Control by HPLC-FD. (**d,e**) MCP-1 (**d**) and IL-8 (**e**): relative concentration in supernatant from 3T3-L1 adipocytes by ELISA. Adipocytes were infected with sh-Control or sh-AAT1 for 4 d, and then stimulated with TNF-α (10 ng/ml) for different times. **(f,g)** MCP-1 (**f**) and IL-8 (**g**): relative concentration in supernatant from 3T3-L1 adipocytes by ELISA. Adipocytes were infected with sh-Control or sh-AAT1 for 4 d, and then stimulated with TNF-α (10 ng/ml) for 2 h. Data are means ± SEM of 6 independent experiments. *P < 0.05 compared with sh-Control group; **P < 0.01 compared with sh-Control group; ^#^P < 0.05 compared with sh-Control + TNF-α group; ^##^P < 0.01 compared with sh-Control + TNF-α group.

Knockdown of AAT1 by lentivirus delivered shRNA was verified by Western blot analysis (Fig. 3a). Consistent with the results of AAT1 expression, the rate of SO_2_ production in 3T3-L1 adipocytes was decreased by AAT1 knockdown (Fig. 3b). HPLC revealed that AAT1 deficiency decreased endogenous SO_2_ levels in comparison to 3T3-L1 adipocytes infected with lentivirus carrying shRNA to Control (sh-Control, Fig. 3c). TNF-α time-dependently upregulated concentrations of MCP-1 and IL-8 in supernatant from both sh-control-infected and sh-AAT1-infected 3T3-L1 adipocytes (Fig. 3d,e). Increased levels persisted for at least 2 h. Of note, concentrations of MCP-1 and IL-8 were greater in supernatant from basal and TNF-α-stimulated 3T3-L1 adipocytes with AAT1 knockdown (Fig. 3f,g).

### Endogenous SO_2_ inhibited TNF-α-induced NF-κB pathway activation in adipocytes

**Figure 4:**
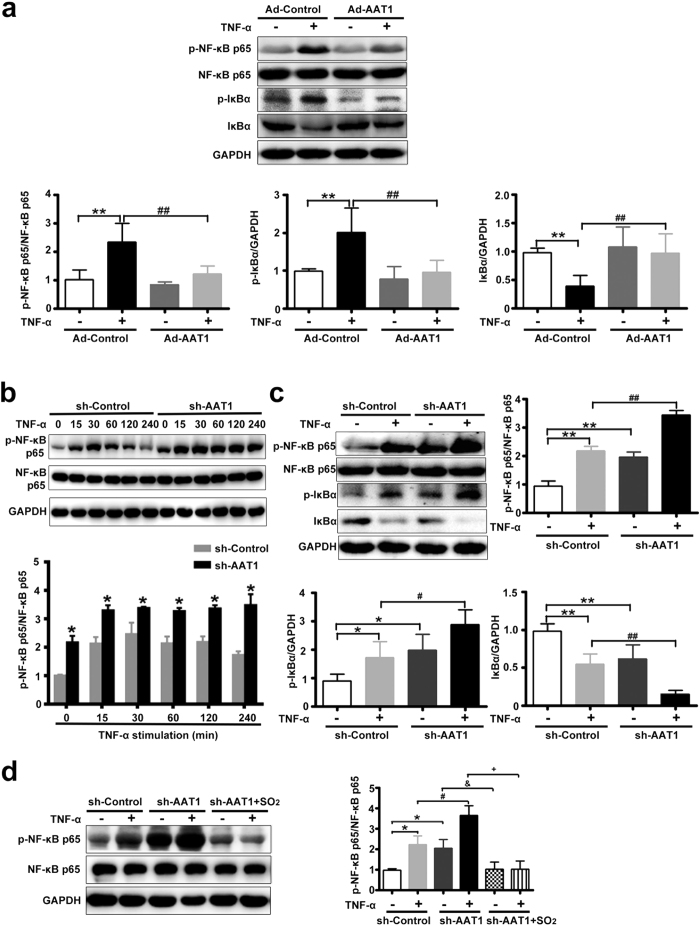
Endogenous SO_2_ inhibited NF-κB pathway activation in TNF-α-stimulated 3T3-L1 adipocytes. (**a**) NF-κB p65 phosphorylation, IκBα phosphorylation and degradation in TNF-α-induced 3T3-L1 adipocytes. Adipocytes were infected with Ad-Control or Ad-AAT1 for 3 d, and then stimulated with TNF-α (10 ng/ml) for 30 min. Data are means ± SEM of 4 independent experiments. **P < 0.01 compared with Ad-Control group; ^##^P < 0.01 compared with Ad-Control + TNF-α group. (**b**) NF-κB p65 phosphorylation in 3T3-L1 adipocytes. Adipocytes were infected with sh-Control or sh-AAT1 for 4 d, and then stimulated with TNF-α (10 ng/ml) for different times. (**c**) NF-κB p65 phosphorylation, IκBα phosphorylation and degradation in TNF-α-stimulated 3T3-L1 adipocytes. Adipocytes were infected with sh-Control or sh-AAT1 for 4 d, and then stimulated with TNF-α (10 ng/ml) for 30 min. (**d**) NF-κB p65 phosphorylation in 3T3-L1 adipocytes. Adipocytes were infected with sh-Control or sh-AAT1 for 4 d, pretreated with SO_2_ derivatives (NaHSO_3_/Na_2_SO_3_, 100 μmol/L) for 1 h, and then stimulated with TNF-α (10 ng/ml) for 30 min. Data are means ± SEM of 4 independent experiments. *P < 0.05 compared with sh-Control group; **P < 0.01 compared with sh-Control group; ^#^P < 0.05 compared with sh-Control + TNF-α group; ^##^P < 0.01 compared with sh-Control + TNF-α group; ^&^P < 0.05 compared with sh-AAT1 group; ^+^P < 0.05 compared with sh-AAT1 + TNF-α group.

The NF-κB pathway plays an important role in regulating inflammation in adipocytes. We then studied the effect of endogenous SO_2_ on the NF-κB pathway during TNF-α-induced inflammatory factors secretion in adipocyte. Western blot analysis revealed that the phosphorylation of NF-κB p65 was increased markedly during stimulation with TNF-α, whereas AAT1 overexpression significantly inhibited NF-κB p65 phosphorylation induced by TNF-α in adipocytes (Fig. 4a).

The activation of NF-κB is processed by the phosphorylation and degradation of IκBα. Western blot analysis revealed that TNF-α treatment increased the phosphorylation of IκBα and induced IκBα degradation, while AAT1 overexpression blocked the effects of TNF-α on IκBα degradation and phosphorylation (Fig. 4a).

On the contrary, a 1.5-fold increase in p65 phosphorylation was observed in response to TNF-α as early as 15 min after TNF-α addition in sh-AAT1-infected adipocytes. Increased levels persisted for at least 2 h. However, the maximal increase in p65 phosphorylation in response to TNF-α was observed at 30 min after TNF-α addition in sh-control-infected 3T3-L1 adipocytes, although it was not as high as that in adipocytes treated with AAT1 knockdown (Fig. 4b). Also, AAT1 knockdown aggravated basal and TNF-α-induced NF-κB p65 phosphorylation, IκBα phosphorylation and its degradation (Fig. 4c). TNF-α binds to TNF-α receptor 1 (TNFR1) or TNF-α receptor 2 (TNFR2), leading to the activation of NF-κB. Herein, we investigated the effect of endogenous SO_2_ on the abundance of TNF-α receptors. The data showed that there was no statistically significant difference in protein expressions of TNFR1 and TNFR2 between adipocytes with AAT1 knockdown and control adipocytes (Supplementary Fig. 1). These results indicated that endogenous SO_2_ inhibited TNF-α-induced NF-κB phosphorylation at least partly in association with suppression of IκBα phosphorylation and degradation.

Western blot analysis demonstrated that 100 μmol/L SO_2_ derivatives could markedly inhibit AAT1 silencing-aggravated NF-κB p65 phosphorylation in basal and TNF-α-treated 3T3-L1 adipocytes (Fig. 4d). These data suggested that SO_2_ exerted a crucially protective role in AAT deficiency-activated NF-κB pathway in 3T3-L1 adipocytes.

### AAT1 deficiency promoted MCP-1 and IL-8 secretion by NF-κB p65 pathway in adipocytes

**Figure 5:**
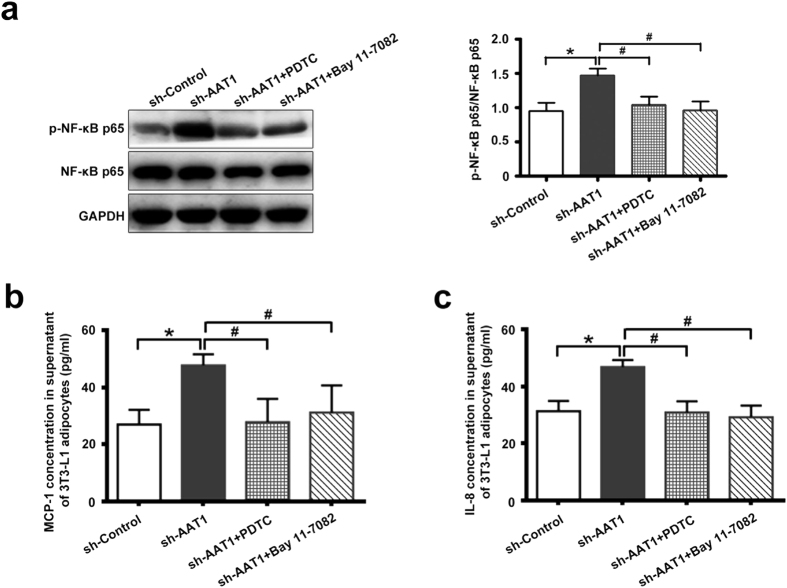
The activation of NF-κB pathway mediated aggravation of MCP-1 and IL-8 secretion in 3T3-L1 adipocytes with AAT1 knockdown. (**a**) NF-κB p65 phosphorylation in 3T3-L1 adipocytes. Adipocytes were infected with sh-Control or sh-AAT1 for 4 d, and then treated with PDTC (10 μmol/L) or Bay 11-7082 (10 μmol/L) for 2 h. (**b–c**) MCP-1 (**b**) and IL-8 (**c**): relative concentration in supernatant from 3T3-L1 adipocytes by ELISA. Adipocytes were infected with sh-Control or sh-AAT1 for 4 d, and then treated with PDTC (10 μmol/L) or Bay 11-7082 (10 μmol/L) for 2 h. Data are means ± SEM of 4 independent experiments. *P < 0.05 compared with sh-Control group; ^#^P < 0.05 compared with sh-AAT1 group.

To further explore whether NF-κB pathway mediated the regulatory role of endogenous SO_2_ in adipocyte inflammation, the effects of pyrrolidine dithiocarbamate (PDTC) and Bay 11-7082, inhibitors of NF-κB activation, on P65 phosphorylation and adipocyte inflammation were observed in AAT1 silencing 3T3-L1 adipocytes. The results showed that by incubating AAT1 silencing cells with PDTC (10 μmol/L) or Bay 11-7082 (10 μmol/L), the phosphorylation of NF-κB p65 was strikingly diminished in AAT1-knockdown 3T3-L1 adipocytes (Fig. 5a), and the concentrations of MCP-1 and IL-8 were reduced as well (Fig. 5b,c).

## Discussion

SO_2_ is a novel gasotransmitter in the cardiovascular system and plays vital roles in regulation of cardiovascular system homeostasis^5^,^6^,^7^. L-cysteine is the major precursor to endogenous SO_2_. It can be metabolized to L-cysteine sulfinate by cysteine dioxygenase (CDO). L-cysteine sulfinate is then metabolized to β-sulfinylpyruvate via AAT1 and AAT2, and finally spontaneously decomposes to pyruvate and SO_2_^5^,^8^,^9^. Some of the endogenous SO_2_ is hydrated to sulfite, whereas the other part stays in the gaseous form^5^,^10^,^11^. Our previous study demonstrated that AAT expression is accompanied by the generation of SO_2_ in several tissues and organs such as the stomach, heart, cerebral gray matters, cerebral white matter, pancreas, lung, kidney, spleen, liver and aorta in mammals^12^. The present study showed that different adipose tissues could also generate endogenous SO_2_, such as epididymal, subcutaneous, perirenal, perivascular and brown adipose tissue.

Two types of isozymes of AAT exist, including AAT1 and AAT2. AAT1 is mainly localized in the cell cytoplasm and AAT2 is localized in the cell mitochondria^12^,^13^. Nowadays, both are found to be present in most cells but erythrocytes from animal tissues^12^,^14^. There are also some differences between AAT1 and AAT2 in terms of functional effects. For example, AAT1 and AAT2 perform different function in the malate-aspartate shuttle^12^. However, until now, we have not understood completely the distribution and function of the SO_2_ generating enzymes in adipose tissues. Thus, the present study indicated that the SO_2_ generating enzymes AAT1 and AAT2 were distributed in adipose tissues of rats detected by real-time PCR, Western blot and immunohistochemical analysis.

Many studies demonstrated that chronic inflammation in adipose tissue was mechanically linked to insulin resistance. Adipose tissue is considered to be an active endocrine organ. Inflammatory cytokines secreted from adipose tissue play vital roles in the development of insulin resistance, obesity and type 2 diabetes^1^,^15^. In particular, pro-inflammatory cytokines MCP-1 and IL-8 are the key regulators of immune cells such as macrophage recruitment, infiltration and activation in adipose tissue in obesity. This process leads to chronic inflammation in adipose tissue and the pathogenesis of obesity and insulin resistance. But the exact interaction between adipocytes and immune cells in the occurrence and development of chronic inflammation still requires further study. Inflammatory cytokines from adipocytes play an important role in the interaction between adipocytes and macrophages, resulting in chronic inflammation. We studied the possible impact of endogenous SO_2_ on MCP-1 and IL-8 secretion in 3T3-L1 adipocytes. The results showed that AAT1 overexpression decreased MCP-1 and IL-8 secretion, whereas AAT1 knockdown increased MCP-1 and IL-8 secretion from TNF-α-stimulated 3T3-L1 adipocytes. These results suggested that endogenous SO_2_ might inhibit secretion of MCP-1 and IL-8 in TNF-α-induced adipocytes, which might play an important role in the protection against adipose inflammation-related diseases such as insulin resistance and obesity.

We then studied how endogenous SO_2_ inhibited MCP-1 and IL-8 secretion. It is widely accepted that nuclear factor-κB (NF-κB) is required for induction of MCP-1 and IL-8 expression and secretion in adipocytes. NF-κB is a key transcription factor in the activation of inflammation in adipocytes. The p65 protein is the key transcriptionally active component of NF-κB, which consists primarily of dimers of the two subunits p50 and p65. IκB, an inhibitory protein in the NF-κB signaling pathway, exists in the cytoplasm in an inactive form associated with the dimer of p65 and p50. During activation of NF-κB, IκB subunit is released from the complex and phosphorylation and translocation of the dimer to the nucleus take place^16^,^17^. In the nucleus, it regulates the transcription of inflammatory genes such as MCP-1 and IL-8. To test the hypothesis that NF-κB was likely to be involved in the underlying mechanism by which endogenous SO_2_ inhibited secretion of MCP-1 and IL-8 in TNF-α-stimulated adipocytes, we first examined the effect of endogenous SO_2_ on the phosphorylation of NF-κB p65 in adipocytes. The results demonstrated that AAT1 overexpression significantly inhibited phosphorylation of NF-κB p65, whereas AAT1 knockdown aggravated it in 3T3-L1 adipocytes stimulated with TNF-α. Furthermore, we found that AAT1 overexpression could suppress the TNF-α-stimulated phosphorylation and degradation of IκBα, which was prevented by AAT1 knockdown, suggesting that endogenous SO_2_ inhibited NF-κB activation in association with preventing IκBα activation. NF-κB inhibitors remarkably antagonized AAT1 deficiency-induced p65 phosphorylation and MCP-1 and IL-8 secretion in 3T3-L1 adipocytes. Thus, these findings implied that the role of SO_2_ in the regulation of adipocyte inflammation was at least partly mediated by NF-κB pathway.

To further investigate whether the effects of AAT manipulation on adipocyte NF-κB pathway activation was caused by SO_2_, we added SO_2_ derivatives to 3T3-L1 adipocytes with AAT1-knock down. The results showed that SO_2_ derivatives at 100 μmol/L significantly inhibited AAT1 silencing-aggravated NF-κB p65 phosphorylation in 3T3-L1 adipocytes with or without TNF-α stimulation, demonstrating that SO_2_ generated by AAT1 played a crucially protective role against adipocyte NF-κB activation in adipocytes with AAT1 knock-down.

TNF-α is one of the primary mediator of the inflammatory response in obesity and insulin resistance. The present study showed that MCP-1 concentration in supernatant from sh-Control-infected and sh-AAT1-infected 3T3-L1 adipocytes increased by 60.62% and 58.98%, respectively, after stimulation with TNF-α for 2 h. And IL-8 concentration in supernatant from sh-Control-infected and sh-AAT1-infected 3T3-L1 adipocytes increased by 65.5% and 44.87%, respectively, after stimulation with TNF-α for 2 h. These findings suggested that it was not an over-sensitization of AAT1 silencing cells to TNF-α. Transducing TNF-α signals through TNF-α receptors resulted in the activation of NF-κB pathway and downstream inflammatory gene transcription. Our data demonstrated that AAT1 deficiency did not affect the protein expressions of two TNF-α receptors, TNFR1 and TNFR2, compared to the control group in the presence or absence of TNF-α.

The limitation of the present study was that we could not exactly define if the effects of bisulfite or sulfite were involved in the gas SO_2_ function. SO_2_ is mainly derived from sulfite (SO_3_^2−^) and bisulfite (HSO_3_^−^) generated after SO_2_ dissolves in cellular cytoplasm^18^. SO_2_ can easily be hydrated to produce sulfurous acid, which subsequently dissociates to form its derivatives, sulfite and bisulfite (3:1 mole ratios in neutral fluids)^19^,^20^,^21^. Therefore, SO_2_, sulfite and bisulfite might exist *in vivo* as a mixture which could not be completely distinguished.

Taken together, our findings suggest that adipose could endogenously synthesize and secrete gasotransmitter SO_2_ which inhibits inflammation via NF-κB pathway in adipocytes. Thus, endogenous SO_2_ is a novel adipocyte-derived inflammatory inhibitor and might be a new therapeutic target for inflammation-related diseases, such as obesity and insulin resistance.

## Methods

### Animal preparation

Animal care and experimental protocols were in accordance with the Animal Management Rule of the Ministry of Health, China. All experimental protocols were approved by the Animal Research Ethics Committee of Peking University First Hospital, Beijing, China (Permit Number: J201335). Eight-week-old male Sprague-Dawley (SD) rats (180–200 g) were obtained from the Experimental Animal Center, Peking University Health Science Center, Beijing, China. The rats were housed under special pathogen-free conditions, at a temperature of 22 °C with 40% humidity and a 12 hour light/12 hour dark cycle.

### Preparation of different tissue samples in rats

Ten male SD rats were anaesthetized using urethane (1 g/kg) by intraperitoneal injection, and then the perivascular adipose tissue, perirenal adipose tissue, epididymal adipose tissue, subcutaneous adipose tissue, brown adipose tissue, heart, lung, liver, spleen, kidney and aorta were harvested rapidly for the following analysis. For SO_2_ determination via PCR and Western blot, samples were frozen and stored in liquid nitrogen. For immunohistochemical analysis, tissues were fixed in 4% polyoxymethylene.

### Determination of SO_2_ concentration of tissue and adipocyte supernatant

The tissues were homogenized in 0.1 mol/L phosphate-buffered saline (PBS, pH 7.4, 10 mL/g tissue), and then centrifuged at 12,000 g for 30 min at 4 °C. The supernatants obtained were prepared for SO_2_ content and protein assay determination. Cell supernatants for SO_2_ concentration determination were taken from TNF-α-stimulated adipocytes.

SO_2_ concentrations were measured using high-performance liquid chromatography with fluorescence detection (HPLC-FD, Agilent 1200 series, Agilent Technologies, Palo Alto, CA, USA)^22^. Briefly, 100 μL of tissue sample or adipocytes supernatant was mixed with 70 μL of 0.212 M/L sodium borohydride in 0.05 M/L Tris-HCl (pH 8.5) and incubated at room temperature for 30 min. The sample was then mixed with 10 μL of 70 mM/L mBrB in acetonitrile, incubated for 10 min at 42 °C, and then mixed with 40 μL of 1.5 M/L perchloric acid. Protein precipitate in the mixture was removed by centrifugation at 12400 × g for 10 min at 23 °C. The supernatant was immediately neutralized by adding 10 μL of 2 M/L Tris-HCl (pH 3.0), and centrifuged at 12400 × g for 10 min. The neutralized supernatant was used for HPLC-FD. Sulfite-bimane was measured by excitation at 392 nm and emission at 479 nm. Quantification was carried out by the standardization of sodium sulfite.

### Determination of aspartate aminotransferase (AAT) 1 and AAT2 mRNA in tissues by real-time PCR

Total RNA in tissues was extracted by Trizol reagent and reverse transcribed by oligo d(T)_18_ primer and M-MuLV reverse transcriptase. Real-time PCR was performed on an ABI PRISM 7300 instrument (ABI USA Sales Corp, Los Angeles, CA, USA). Samples and standard DNA were determined in duplicate. The PCR condition was predenaturing at 95 °C for 5 min, then 95 °C for 15 s, and 60 °C for 1 min for 35 cycles. The amount of β-actin cDNA in the sample was used to calibrate the sample amount used for the determination. The PCR products were separated in agarose gel electrophoresis.

Sequences of the primers and TaqMan probes were provided as follows. For AAT1, forward, 5′-CCAGGGAGCTCGGATCGT-3′, reverse, 5′-GCCATTGTCTTCACGTTTCCTT-3′, TaqMam probe, 5′-CCACCACCCTCTCCAACCCTGA-3′, the product size is 79 bp. For AAT2, forward, 5′-GAGGGTCGGAGCCAGCTT-3′, reverse, 5′-GTTTCCCCAGGATGGTTTGG-3′, TaqMan probe, 5′-TTTAAGTTCAGCCGAGATGTCTTTC-3′, the product size is 82 bp; for β-actin, forward, 5′-ACCCGCGAGTACAACCTTCTT-3′, reverse, 5′-TATCGTCATCCATGGCGAACT-3′, TaqMan probe, 5′-CCTCCGTCGCCGGTCCACAC-3′, the product size is 80 bp.

### Determination of AAT, NF-κB and IκBα protein expression in tissues and adipocytes by Western blot analysis

The different rat tissues were homogenized and lysed. The adipocytes were harvested and lysed. Equal amounts of proteins were boiled and separated by SDS-PAGE and transferred onto nitrocellulose membranes. The following primary antibodies were used: anti-AAT1 (Sigma-Aldrich, St. Louis, MO, USA), anti-AAT2 and anti-GAPDH (Kangcheng, Shanghai, China), anti-p-NF-κB, anti-NF-κB, anti-p-IκBα and anti-IκBα (Cell Signaling Technology, Danvers, MA, USA). HRP-labeled Sigma-Aldrich goat anti-mouse and Sigma-Aldrich goat anti-rabbit at dilutions of 1:6000 and 1:4000 were used as secondary antibodies. The bands were visualized using Enhanced Chemiluminescence Detection Kit (Thermo Scientific, Rockford, IL, USA). The densitometric analysis of the positive bands was performed by use of AlphaImager (San leandro, CA, USA).

### Expression of AAT1 and AAT2 in different tissues using immunohistochemical analysis

We detected the expression of AAT1 and AAT2 in different tissues by using immunohistochemical analysis. Sections of tissues were dewaxed by dimethylbenzene, washed three times with PBS, and then treated with 3% H_2_O_2_ for 10 min. The slides were blocked with 5% bovine serum albumin (BSA) working fluid at 37 °C for 30 min, and incubated overnight at 4 °C with AAT1 and AAT2 antibodies (dilutions of 1:25, respectively). Slides were then washed with PBS. Biotinylated anti-rabbit or anti-mouse IgG was incubated for 60 min at 37 °C. After the slides were rinsed in PBS three times, the sections were stained with 3,3′-diaminobenzidine (DAB) to develop color. The sections were dehydrated and mounted. Positive signals were defined as brown granules in tissues under light microscopy. For negative controls, primary incubation was performed with non-immune goat serum instead of primary antibodies.

### 3T3-L1 cell culture

Murine 3T3-L1 cell line was obtained from the American Type Culture Collection (ATCC, Manassas, VA, USA). The 3T3-L1 preadipocytes were cultured in Dulbecco’s modified Eagle’s medium (DMEM) supplemented with 10% FBS, 2 mmol/L glutamine and 20 mmol/L HEPES (pH 7.4) in a humidified atmosphere of 5% CO_2_ at 37 °C. After reaching 100% confluence, 3T3-L1 preadipocytes were stimulated to differentiate using a differentiation mixture containing 0.25 μM dexamethasone, 0.5 mM IBMX and 1 μM insulin in DMEM with 10% FBS. After 2 days, the medium was replaced with DMEM supplemented with 10% FBS and 1 μM insulin. Cultures were incubated for 2 days; afterwards the culture medium was replaced again with DMEM supplemented with 10% FBS and replaced at 2 day intervals until the adenovirus or lentivirus infection was performed on days 6-8^23^,^24^,^25^.

### 3T3-L1 cell treatment

The adenovirus containing the cDNA encoding AAT1 was purchased from Vigene Biosciences (Shandong, China). The lentivirus carrying shRNA to AAT1 was purchased from Pregene (Beijing, China). 3T3-L1 adipocytes were transduced at a multiplicity of infection of 100 PFU/cell for 24 h^21^. Transduced cells were incubated for 48–72 h at 37 °C in 5% CO_2_, followed by a stimulation with TNF-α (10 ng/ml) for 30 min or 2 h.

### Determination of SO_2_ production

The different rat tissue homogenate was prepared, then mixed with the reaction buffer including 100 mmol/L potassium phosphate buffer (pH 7.4), 10 mmol/L L-cysteine and 2 mmol/L pyridoxal 5′-phosphate. The mixture was incubated at 37 °C for 90 min. For the production of SO_2_ in adipocytes, at the end of treatment, cell medium was replaced with fresh medium containing 10 mmol/L L-cysteine and 2 mmol/L pyridoxal 5′-phosphate, and then, the cells were incubated for further 4 h. Released SO_2_ was detected by HPLC-FD. The rate of SO_2_ production is expressed as nmol SO_2_ formed from 1 g of protein per minute (nmol/min/g protein) determined by the Bradford assay.

### Statistical analysis

Results are expressed as means ± SEM. The analysis was done using GraphPad Prism 5. For multiple group comparisons, ANOVA followed by a *post-hoc* analysis (Newman-Keuls test) was used. P < 0.05 were considered statistically significant.

## Additional Information

**How to cite this article**: Zhang, H. *et al.* Endogenous sulfur dioxide is a novel adipocyte-derived inflammatory inhibitor. *Sci. Rep.* **6**, 27026; doi: 10.1038/srep27026 (2016).

## Supplementary Material

Supplementary Information
