# Supplementary material for: Endogenous sulfur dioxide is a novel adipocyte-derived inflammatory inhibitor
Source: Sci Rep. 2016 Jun 1;6:27026. doi: 10.1038/srep27026 (PMC4887903; doi:10.1038/srep27026)
Supplement: Supplementary Information [file srep27026-s1.doc]

**Endogenous sulfur dioxide is a novel adipocyte-derived inflammatory inhibitor**

Heng Zhang ^1^*, Yaqian Huang ^2^*, Dingfang Bu ^3^, Selena Chen ^4^, Chaoshu Tang ^5,6^, Guang Wang ^1#^, Junbao Du ^2,6#^, Hongfang Jin ^2#^

**SUPPLIMENTAL FIGURES**


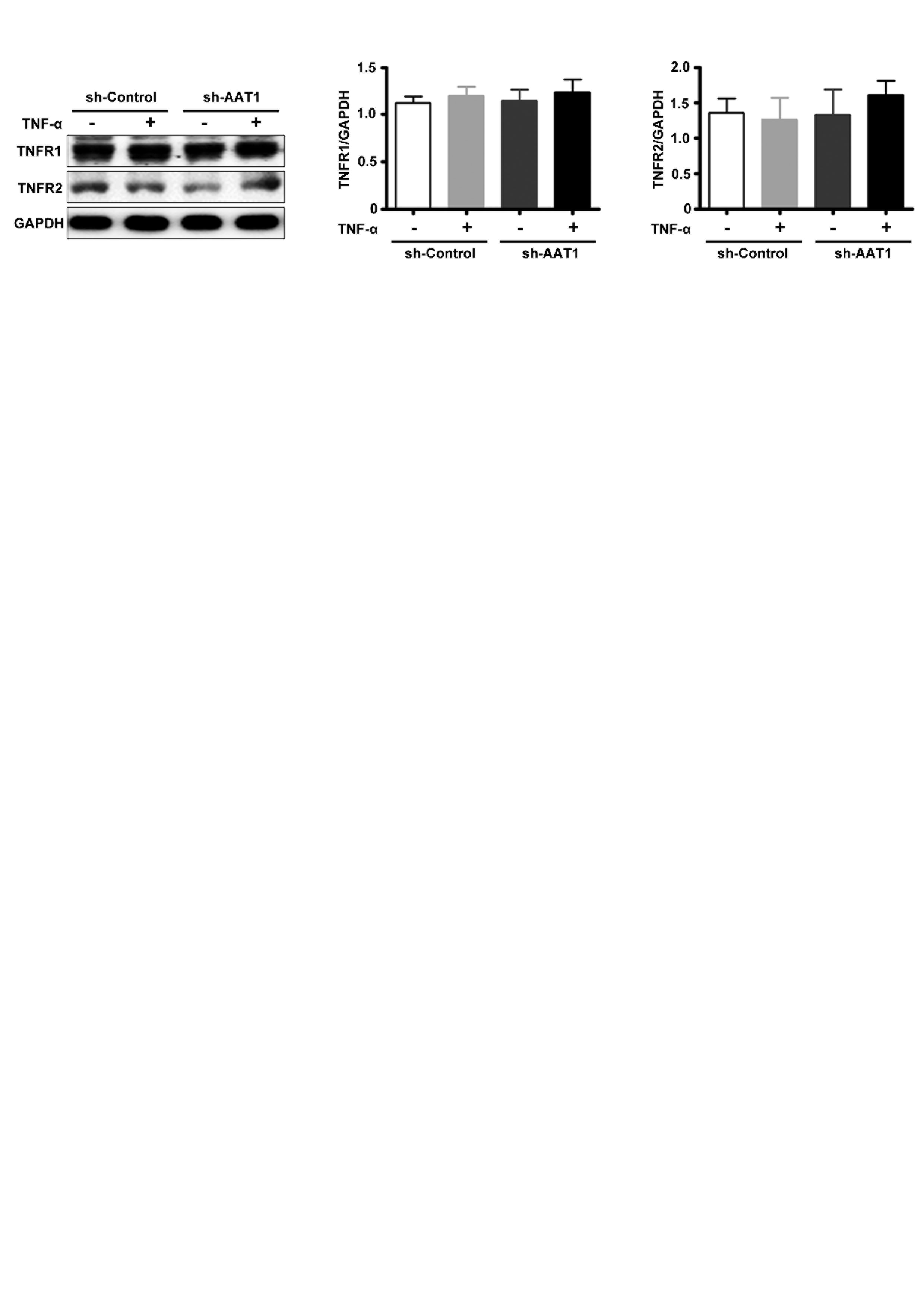


**Supplementary Figure 1. AAT1 deficiency did not affect protein expressions of TNF-α receptors in 3T3-L1 adipocytes.** Representative Western blot and quantification of TNFR1 and TNFR2 in 3T3-L1 adipocytes. Adipocytes were infected with sh-Control or sh-AAT1 for 4 d, and then stimulated with TNF-α (10 ng/ml) for 2 h.
